# Spinal Serotonergic and Opioid Receptors Are Involved in Electroacupuncture-Induced Antinociception at Different Frequencies on ZuSanLi (ST 36) Acupoint

**DOI:** 10.1155/2013/291972

**Published:** 2013-03-28

**Authors:** Chi-Chung Kuo, Huei-Yann Tsai, Jaung-Geng Lin, Hong-Lin Su, Yuh-Fung Chen

**Affiliations:** ^1^Department of Neurology, Tzu Chi General Hospital, Taichung Branch, Taichung 42743, Taiwan; ^2^Department of Medicine, College of Medicine, Tzu Chi University, Hualien 97002, Taiwan; ^3^Department of Life Sciences, National Chung Hsing University, Taichung 40227, Taiwan; ^4^Department of Pharmacy, China Medical University Hospital, Taichung 40447, Taiwan; ^5^Department of Chinese Medicine, College of Chinese Medicine, China Medical University, Taichung 40402, Taiwan; ^6^Department of Pharmacology, College of Medicine, China Medical University, 91 Hsueh-Shih Road, Taichung 40402, Taiwan

## Abstract

The present study was conducted to evaluate the effect of electroacupuncture-(EAc-) induced antinociception (EAA) at different currents and frequencies in rat spinal cord. We found that naloxone (0.05 **μ**g i.t.) blocked EAA at different frequencies. Naltrindole (0.05 **μ**g i.t.) blocked EAA on the 7th day after EAc of 100 Hz. 5,7-Dihydroxytryptamine (100 **μ**g i.t.) significantly inhibited EAA at different frequencies on the 7th day after EAc. Pindobind (0.5 **μ**g i.t.), a 5-HT_1A_ antagonist, notably attenuated EAA at different frequencies. Ketanserin (0.5 **μ**g i.t.), inhibited EEA at a lower frequency (<10 Hz) than at a higher frequency (100 Hz). LY-278584 (0.5 **μ**g i.t.) significantly inhibited EAA at a higher frequency (100 Hz) on the 7th day after EAc. The direction of effect of 8-OH-DPAT, on EAA was dependent on dosage. It had an inhibitory effect at a low dose (0.5 **μ**g i.t.) and a high frequency (100 Hz) but enhanced EAA at a higher dose at lower frequencies (<10 Hz). DOI (10 **μ**g, i.t.), did not affect EAA. These data indicate that the mechanism of EAA involves opioid receptors, and the serotonergic system, particularly, **μ**-, **δ**-opioid and 5-HT_1A_, 5-HT_3_ receptors and it is also dependent on the EAc frequency.

## 1. Introduction 

Acupuncture, a traditional Chinese medicine, has been used to relieve pain for more than 2000 years, and it has been used in over 160 countries. Acupuncture has been proposed by an NIH consensus committee as a complementary medicine [1]. Treatment efficacy of acupuncture has been acknowledged worldwide. The physiological and biochemical mechanisms underlying acupuncture analgesia have been receiving increasing attention. 

Analgesia by peripheral nerve stimulation, either transcutaneous nerve stimulation (TENS), acupuncture, or electroacupuncture (EAc), was demonstrated in anesthetized monkeys and in rodents [2–4]. In the spinal cord, substance P released by A*δ* and C fiber while nociception entering the spinal cord posterior horn was blocked by naloxone. Not only substance P but also endorphin, encephalin, and dynorphin could be induced in the spinal cord. *β*-Endorphin predominantly synthetized in the arcuate nucleus of the hypothalamus has major analgesic effects via *μ*-, *κ*-opioid receptors in the periaqueductal gray region [5]. Enkephalins are ligands of both *μ* and *δ* receptors [6–8]. Dynorphin is a relatively specific ligand for *κ* receptors in the spinal cord of the rat [9]. 

Electroacupuncture antinociception (EAA) induced by low frequency may be mediated by endorphins. Effect of high frequency stimulation is not mediated by endorphin but may be due to either serotonin or dynorphins in the spinal cord [10]. In previous studies, it was shown that 5-HT release from the spinal dorsal horn was significantly stimulated by somatostatin and substance P *in vitro*, but not by neurotensin or met-enkephalin [11]. The influence of EAc on serotonin release may cause activation of enkephalin-interneurons which presynaptically inhibit the primary sensory neurons in the spinal cord [12].

On the other hand, studies have shown that EAc-induced analgesia can be blocked by opioid receptor antagonists in human and animals [13–16]. One interpretation of those results is that an opioid mechanism is involved in mediating EAA. Antibody microinjection studies showed that 2/15 Hz EAA could be blocked by intrathecal (i.t.) injection of any one of the three categories of antibodies directed to met-enkephalin and leu-enkephalin [17], dynorphin A [18], and dynorphin B [9]. Moreover, different frequencies of EAc may be mediated by specific opioid receptors [19, 20].

Lumbar catheterization of the subarachnoid space in the spine is commonly used to study the rat spinal cord [21, 22]. The method (A-O method) involves freeing neck muscle from the occipital crest and sliding the catheter through a slit in the exposed atlanto-occipital (A-O) membrane, and caudally along the spinal cord [22]. Disadvantages of the A-O method are that some animals die during the first days after catheterization (3%–5%) and animals show signs of neurological impairment after implantation (10%–30%) [23–25]. In the present study, the operation procedure modified from Tsai et al. [11] was used to perform the intrathecal catheterization for drugs treatment when studying EAA.

## 2. Materials and Methods

### 2.1. Animals

 Male Wistar rats weighing 240 to 260 g were purchased from National Taiwan University College of Medicine Laboratory Animal Center (NTU CMLAC). Animals were allowed at least 1 week of adaptation before the experiments, and they had free access to food and water. The laboratory had a 12 hr/12 hr light/dark cycle. The room temperature was controlled at 22 ± 1°C. The experimental protocol was approved by the Institutional Animal Care and Use Committee (IACUC), China Medical University, Protocol 101–250.

### 2.2. Drugs

 Opioid receptor antagonists: naloxone, naltrindole (*δ*-opioid antagonist); serotonin neurotoxin: 5,7-dihydroxytryptamine (5,7-DHT); serotonin antagonists: pindobind (PDB, 5-HT1A antagonist), ketanserin tartrate (5-HT_2_ antagonist), LY-278584 maleate (5-HT_3_ antagonist), R(+)-8-hydroxy-dipropylaminotetralin (8-OH-DPAT, 5-HT_1A_ agonist), R(+)-2,5-dimethoxy-4-iodoamphetamine HCl (DOI, 5-HT_2/1C_ agonist), 2-methylserotonin maleate (2-methyl-5-HT, 5-HT_3_ agonist), all the aforementioned were from RBI Co., USA. Drugs were dissolved in artificial CSF (vehicle). The artificial CSF (ACSF) is a Krebs-bicarbonate solution (compositions: NaCl 120 mM, KCl 5 mM, NaHCO_3_ 15 mM, MgSO_4_ 1 mM, CaCl_2_ 1.5 mM, and glucose 10 mM).

### 2.3. Intrathecal Catheterization

**Figure 1:**
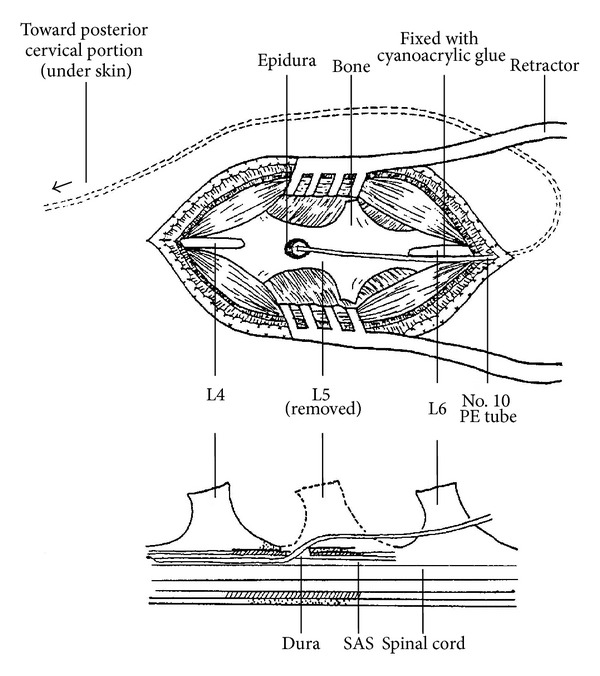
Diagram of intrathecal cannulation. A middle lumbar skin incision was performed, following the paravertebral muscle detached from the spinal process and retracted laterally. L5 spinal process was removed then opened the dura for exposing the spinal cord. The dura was perforated with a short bevel of no. 30 gauze needle following inserted 2 cm PE-10 into the subarachnoid space (SAS). A drop of cyanoacrylic glue was added for anchoring the PE-10 besides the L6 spinal process. The operation wound was irrigated with normal saline and closed in layers with silk streaks, no. 4.

 The operation procedures were modified from Tsai et al. [11]. The skin over the posterior cervical and lumbar region was shaved and prepared with povidone iodine (betadine). Rats were anesthetized with ether. The fifth spinal process was removed and the dura mater exposed. The dura was perforated with a short bevel no. 30-gauze needle, resulting in some leakage of CSF. A 16 cm in length polyethylene catheter (PE-10, i.d. 0.28 mm) previously filled with artificial CSF (ACSF) was immediately inserted 2 cm tangentially through the dura opening into the subarachnoid space, then anchored at the sixth spinal process with cyanoacrylic glue. The wound was irrigated with normal saline and closed in layers with silk streaks (no. 4). The left catheter was buried under the skin and the tip of catheter was threaded throughout the posterior cervical skin, also fixed with cyanoacrylic glue and tightened with silk threads (Figure 1).

### 2.4. The Acupoint, Electroacupuncture (EAc), and Tail-Flick Test

 The procedure was carried out initially at 3 hrs after the rat recovered from ether anesthesia postintrathecal (i.t.) cannulation. Rats were placed in a transparent cylinder holder without body restriction. Acupuncture was performed by inserting fine stainless acupuncture needles (no. 36, 0.2 mm in diameter) at the bilateral acupoints ZuSanLi (ST36), 5 mm below the knee and 2 mm lateral to the tibia with 5 mm in depth. Electroacupuncture was applied with different currents (1 mA, 2 mA, and 3 mA) and different frequencies (2 Hz, 10 Hz, and 100 Hz) for 10 minutes using an electric stimulator (Coulbourn, C13-65). 1 mA current was chosen for the subsequent experiments in which drugs administration was held before electroacupuncture in this study.

 The rat was placed in an acrylic holder to adapt for at least 15 minutes at ambient room temperature controlled at 22 ± 1°C. The pain threshold was determined by a tail-flick Analgesia Meter (Muromachi Kikai Co. MK-330). The nociceptive tail-flick (TF) reflex was evoked by noxious radiant heat (0.8 mm in diameter) by a 50 W projector lamp applied to the underside of the tail at 1 cm apart, with the distal site 2-3 cm from the end of the tail. TF latency was measured by a photocell timer circuit from the opening of a shutter until the rat withdrew its tail from the heat source. Intensity was set such that baseline TF latencies were typically between 2 and 4 seconds. The cut-off value of tail-flick latency was not over 10 seconds to avoid damaging the skin. 

### 2.5. Experimental Protocol

**Figure 2:**
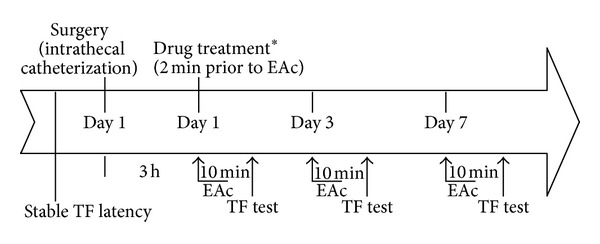
Schedule of drug treatment and experiment orders. Stable TF latency was carried out firstly before the surgery. EAc was performed 3 hrs after the rat recovered from ether anesthesia postintrathecal (i.t.) cannulation. Acupuncture was performed by inserting fine stainless acupuncture needles at bilateral acupoints ZuSanLi (ST36). EAc was applied with different currents (1 mA, 2 mA, and 3 mA) and different frequencies (2 Hz, 10 Hz, and 100 Hz) for 10 min, which was performed with electric stimulator. 1 mA current was chosen for the subsequent experiments in which drugs administration was held before electroacupuncture in this study. The pain threshold was determined by tail-flick Analgesia Meter. The basal TF latencies were typically between 2 and 4 seconds. EAc: electroacupuncture; *TF: tail-flick; 5,7-DHT: 5,7-dihydroxytryptamine, one week prior to EAc.

 The stable baseline TF latency was established in each experiment before intrathecal (i.t.) catheterization. Rats that exhibited neurological deficits or motor dysfunction following recovery from anesthesia after i.t. catheterization (described earlier) were sacrificed. The drugs were administered 2 minutes before electroacupuncture (except 5,7-DHT administered one week before experiments). 10 *μ*L of drug or artificial CSF (vehicle) was injected via i.t. within 30 seconds, followed by flushing 10 *μ*L of artificial CSF. The TF reflex latency was measured at 0, 15, 30, 60, 90, 120, and 150 minutes after a 10 min electroacupuncture. The same procedures (drugs administration, electroacupuncture, and TF latency measurement) were repeated on the 1st, 3rd, and 7th days after intrathecal catheterization was performed. Data for changes in TF latency are presented as percentage (%) change of pain threshold = [TF latency (after EAc) − baseline TF latency (before EAc)] × 100/[baseline TF latency (before EAc)]. The experimental protocol is shown in Figure 2.

### 2.6. Data and Statistical Analysis

 Data were expressed as mean ± standard error (SE) and analyzed using one-way analysis of variance (ANOVA), followed by Scheffe's test. When the probability (*P*) was less than or equal to 0.05, differences were considered significant.

## 3. Results

### 3.1. The Effects of Electroacupuncture (EAc) at Different Frequencies on the 1st, 3rd, and 7th Days

**Figure 3:**
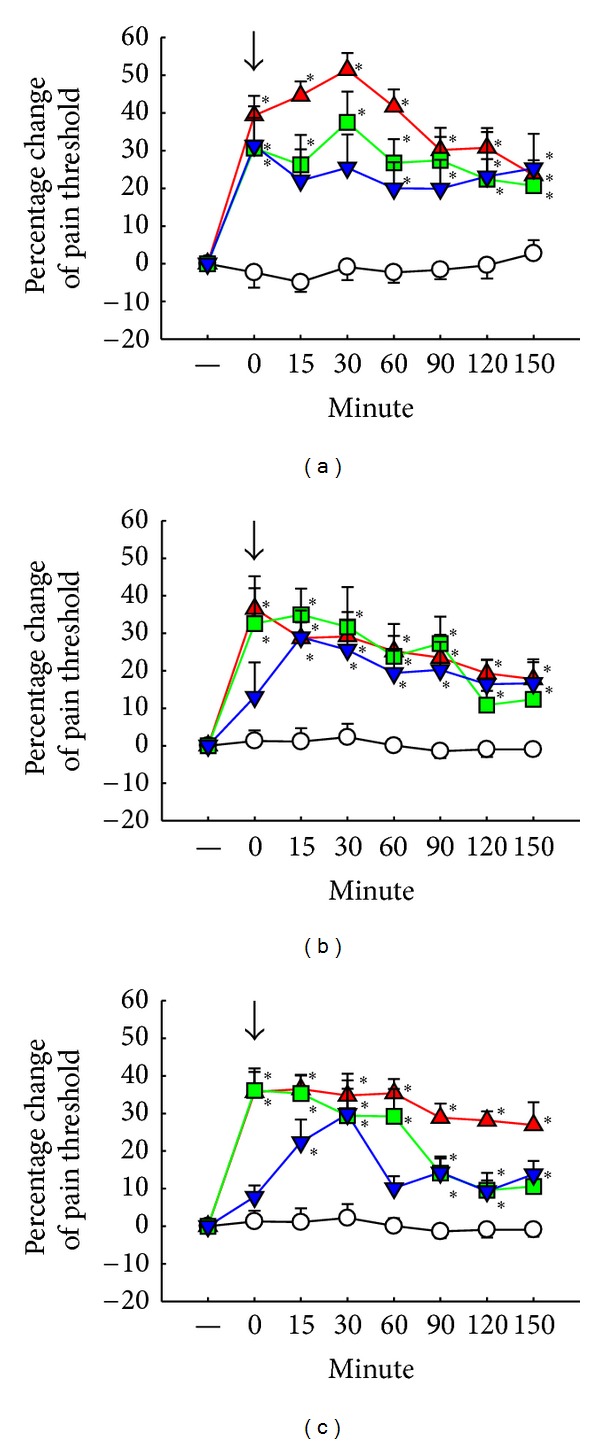
Antinociceptive effects of electroacupuncture (EAc) in different frequencies on the 1st, 3rd and 7th days after intrathecal cannulation by tail-flick test. ↓: initiation point of EAc except sham group. ○: sham group. ▲: 2 Hz, 1 mA. *▼*: 10 Hz, 1 mA. ■: 100 Hz, 1 mA. (a) Determined on 1st day; (b) determined on 3rd day; (c) determined on 7th day (described as text). Data are shown as mean ± S.E. *P < 0.05 compared with sham group (*n* = 10).

The pain threshold increased by 51.41% in 30 minutes on the 1st day after 2 Hz, 1 mA EAc (Figure 3(a)). The antinociceptive effect occurred until 150 minutes after EAc. The pain threshold increased by 29.16% and 34.76% on the 3rd and 7th days, respectively. The duration of EAA revealed on the 3rd and 7th days was similar to the effect on the 1st day after intrathecal cannulation (Figures 3(b) and 3(c)). We also examined the effect of EAc at 2 mA and 3 mA. The pain threshold increased by 23.65% and 37.47% in 30 minutes and up to 150 min. Data on different currents and frequencies on the 1st, 3rd, and 7th days are shown in Table 1. 

### 3.2. Effects of Naloxone and Naltrindole on EAc at Different Frequencies

**Figure 4:**
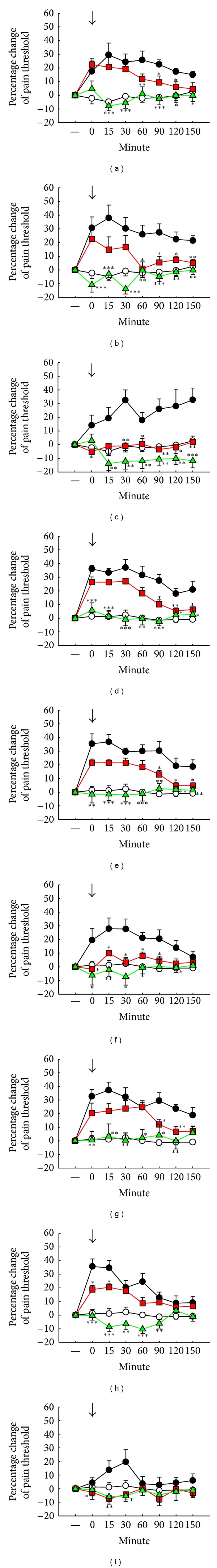
Pretreatment with opioid antagonists influences EAc-induced antinociception in different frequencies on the 1st (a, b, c), 3rd (d, e, f), and 7th (g, h, i) days after intrathecal cannulation. (a), (d), (g): 1 mA, 2 Hz EAc; (b), (e), (h): 1 mA, 10 Hz EAc; (c), (f), (i): 1 mA, 100 Hz EAc; ↓: initiation point of EAc except sham group. ○: sham group. *⚫*: ACSF: artificial CSF. ▲: naloxone (0.05 *μ*g/10 *μ*L, i.t.). ■: naltrindole (0.05 *μ*g/10 *μ*L, i.t.). Data are shown as mean ± S.E. *P < 0.05, **P < 0.01, and ***P < 0.001 compared to the ACSF group (*n* = 10).

Pretreatment with naloxone (0.05 *μ*g/10 *μ*L, i.t.), a *μ*-opioid antagonist, completely blocked the EAc-induced antinociception (EAA) at three different frequencies of EAc on the 1st, 3rd, and 7th days (Figures 4(a)–4(i)). Naltrindole (0.05 *μ*g/10 *μ*L, i.t.), a *δ*-opioid antagonist, significantly inhibited EAA at high (100 Hz) frequency on the 1st, 3rd, and 7th days (Figures 4(c), 4(f), and 4(i)). 

### 3.3. Effects of 5,7-Dihydroxytryptamine (5,7-DHT) Pretreatment on EAc at Different Frequencies

**Figure 5:**
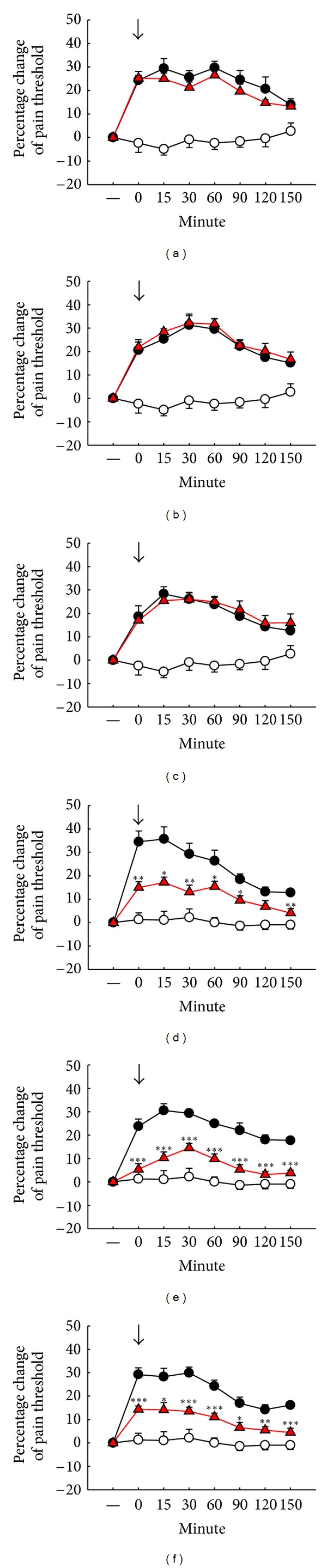
Pretreatment with 5,7-DHT influences EAc-induced antinociception in different frequencies on the 1st (a, b, c) and 7th (d, e, f) days. (a), (d): 1 mA, 2 Hz EAc; (b), (e): 1 mA, 10 Hz EAc; (c), (f): 1 mA, 100 Hz EAc; ↓: initiation point of EAc except sham group. ○: sham group. *⚫*: ACSF (artificial CSF). ▲: 5, 7-DHT (5,7-dihydroxy tryptamine, serotonin neurotoxin, 100 *μ*g/10 *μ*L, i.t.). Data are shown as mean ± S.E. *P < 0.05, **P < 0.01, and ***P < 0.001 compared to the ACSF group (*n* = 10).

The pain threshold of EAc was not affected by 5,7-DHT (100 *μ*g/10 *μ*L) on the 1st day at different EAc frequencies of (Figures 5(a)–5(c)). Until one week after treatment with 5,7-DHT, the pain threshold of EAc was significantly inhibited by 5,7-DHT on the 7th day at three different frequencies (Figures 5(d)–5(f)). 

### 3.4. Effects of 5-HT Antagonists on EAc at Different Frequencies

**Figure 6:**
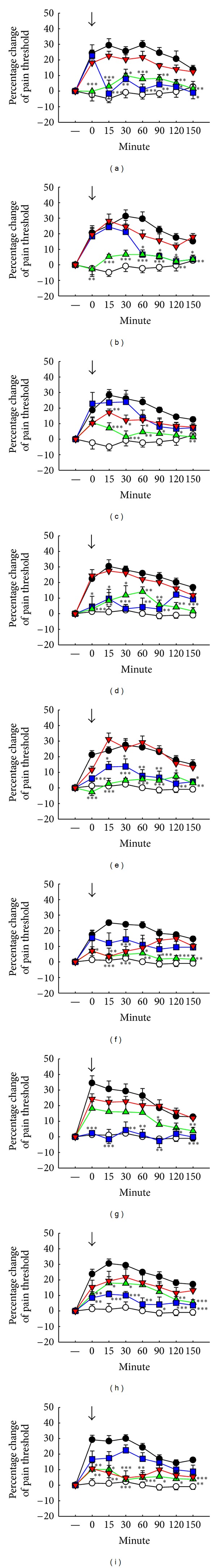
Pretreatment with 5-HT antagonist influences EAc-induced antinociception in different frequencies on the 1st (a, b, c), 3rd (d, e, f), and 7th (g, h, i) days. (a), (d), (g): 1 mA, 2 Hz EAc; (b), (e), (h): 1 mA, 10 Hz EAc; (c), (f), (i): 1 mA, 100 Hz EAc, ↓: initiation point of EAc except sham group. ○: sham group; *⚫*: ACSF (artificial CSF). ▲: PDB: (pindobind, 5-HT_1A_ antagonist, 0.5 *μ*g/10 *μ*L, i.t.). ■: KTS (ketanserin, 5-HT_2_ antagonist 0.5 *μ*g/10 *μ*L, i.t.). *▼*: LY-278584 (5-HT_3_ antagonist, 0.5 *μ*g/10 *μ*L, i.t.). Data are shown as mean ± S.E. *P < 0.05, **P < 0.01, and ***P < 0.001 compared to the ACSF group (*n* = 10).

Figure 6 shows effects of pretreatment with 5-HT antagonists on EAc. Pindobind-5-HT_1A_ (PDB, 5-HT_1A_ antagonist, 0.5 *μ*g/10 *μ*L, i.t.) markedly blocked EAA at different frequencies on the 1st, 3rd, and 7th days (Figures 6(a)–6(i)). Pretreatment with ketanserin (5-HT_2_ antagonist, 0.5 *μ*g/10 *μ*L, i.t.) reduced EAA at a lower frequency (<10 Hz) of EAc on the 1st, 3rd, and 7th days (Figures 6(a), 6(b), 6(c), 6(d), 6(g), and 6(h)). LY-278584 (5-HT_3_ antagonist, 0.5 *μ*g/10 *μ*L) significantly inhibited high frequency EAA on the 1st, 3rd, and 7th days (Figures 6(c), 6(f), and 6(i)). 

### 3.5. Effects of 5-HT_1A_ Agonist, 8-OH-DPAT on EAc at Different Frequencies

**Figure 7:**
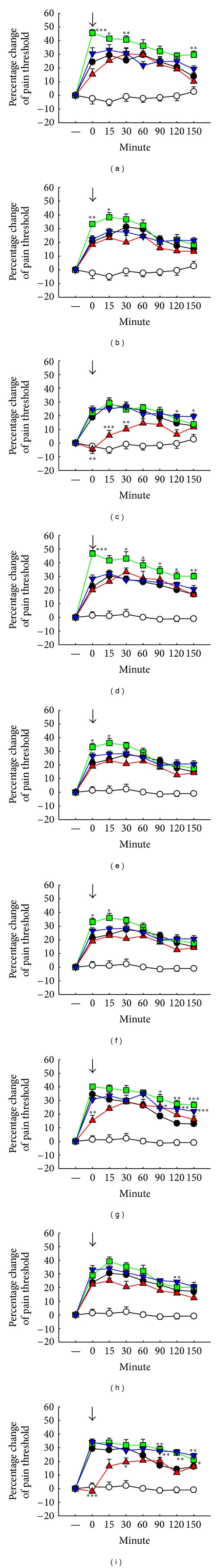
Pretreatment with 8-OH-DPAT influences EAc-induced antinociception in different frequencies on the 1st (a, b, c), 3rd (d, e, f), and 7th (g, h, i) days after intrathecal cannulation. (a), (d), (g): 1 mA, 2 Hz EAc; (b), (e), (h): 1 mA, 10 Hz EAc; (c), (f), (i): 1 mA, 100 Hz EAc, ↓: initiation point of EAc except sham group. ○: sham group. *⚫*: ACSF (artificial CSF). ▲: 8-OH-DPAT (DPAT, 5-HT_1A_ agonist, 0.5 *μ*g/10 *μ*L, i.t.). ■: DPAT (1 *μ*g/10 *μ*L, i. t.); *▼*: DPAT (2 *μ*g/10 *μ*L, i.t.). Data are shown as mean ± S.E. *P < 0.05, **P < 0.01, and ***P < 0.001 compared to the ACSF group (*n* = 10).

 8-OH-DPAT (DPAT), a 5-HT_1A_ agonist, inhibited EAA which was dependent on DPAT dose and EAc. DPAT (0.5 *μ*g/10 *μ*L, i.t.) inhibited the EAA at a high frequency (100 Hz) of EAc on the 1st, 3rd, and 7th days (Figures 7(c), 7(f), and 7(i)). However, a concentration of DPAT greater than 1 *μ*g/10 *μ*L (i.t.) potentiated the EAA at a lower frequency (<10 Hz) of EAc (Figures 7(a), 7(b), 7(d), 7(e), and 7(g)). 

### 3.6. Effects of 5-HT_2_ and 5-HT_3_ Agonists on the EAc at Different Frequencies

**Figure 8:**
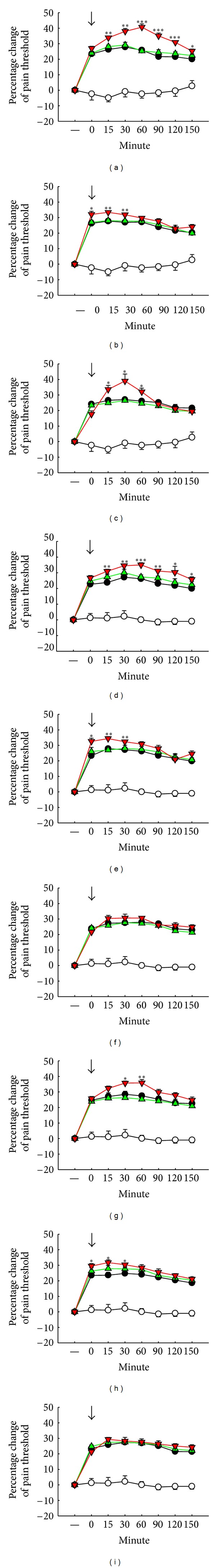
Pretreatment with 5-HT agonists influences EAc-induced antinociception in different frequencies on the 1st (a, b, c), 3rd (d, e, f), and 7th (g, h, i) days after intrathecal cannulation. (a), (d), (g): 1 mA, 2 Hz EAc; (b), (e), (h): 1 mA, 10 Hz EAc; (c), (f), (i): 1 mA, 100 Hz EAc; ↓: initiation point of EAc except sham group. ○: sham group. *⚫*: ACSF (artificial CSF). ▲: DOI (R(+)-2,5-dimethoxy- 4-iodoamphetamine HCl, 5-HT_2/1C_ agonist, 10 *μ*g/10 *μ*L, i.t.). *▼*: 2-methyl-5-HT (2-methylserotonin maleate, 5-HT_3_ agonist, 50 *μ*g/10 *μ*L, i.t.). Data are shown as mean ± S.E. *P < 0.05, **P < 0.01, and ***P < 0.001 compared to the ACSF group (*n* = 10).

 R(+)-2,5-dimethoxy-4-iodoamphetamine HCl (10 *μ*g/10 *μ*L, i.t.), a 5-HT_2/1C_ agonist, did not significantly affect EAA at different frequencies. However, pretreatment with 2-methy-5-HT (50 *μ*g/10 *μ*L, i.t.), a 5-HT_3_ agonist, enhanced EAA at a lower frequency (<10 Hz) (Figures 8(a), 8(b), 8(d), 8(e), 8(g), and 8(h)). 

## 4. Discussion

There has been increasing attention given to the use of EAc for treating pain both experimentally and clinically. Low and high electrical frequencies are an important component of EAc. A very low frequency of EAc at 0.4 Hz did not produce a desired analgesic effect, whereas 4 Hz or 200 Hz EAc could induce considerable analgesia [10]. In the present study, the relative lower frequencies of EAc (<10 Hz) may provide more stable and longer duration of antinociception when compared with a high EAc frequency (100 Hz). Similar results were present in another study that applied 2 Hz or 100 Hz EAc [26]. We found that EAA could be reobtained on different days in the present study. Similarly, repeated electroacupuncture had a cumulative effect on analgesia that may be associated with regulation of the hypothalamus-pituitary axis [27].

Earlier studies reported that *μ*- and *δ*-receptors were involved in EAc analgesia at a low frequency (2/15 Hz) [19, 20]. In the present study, the administration of drugs was intrathecally directed into the rat spinal cord. Effect of drugs on EAA was determined by the tail-flick test. Naloxone, a *μ*-antagonist, completely abolished the EAA at different electrical frequencies. However, the *δ*-receptor participated in the EAA when the frequency of EAc was more than 100 Hz. The results were similar to a previous report [28]. Data from the present study suggested that EAA occurred via *μ*-opioid receptors at a low frequency (<10 Hz) and that activation of *δ*-opioid receptors was at a high frequency (100).

The present study also examined the role of the serotonergic pathway in mediating effect of EAc. EAA was blocked by pindobind, a 5-HT_1A_ antagonist, and by ketanserin, a 5-HT_2_ antagonist at a low frequency (<10 Hz), and by LY-278584, a 5-HT_3_ antagonist at a high frequency (100 Hz). On the other hand, EAA was potentiated by DPTA, a 5-HT_1A_ agonist at a high dose (>1 *μ*g), and by 2-methyl-5-HT, a 5-HT_3_ agonist at a low frequency (<10 Hz). Therefore, the effect of DPTA on EAA was dependent on drug dosage. It has been suggested that DPTA could decrease the turnover rate of 5-HT in presynaptic serotonergic neurons at a low dose (0.05 mg/kg, s.c.) and stimulate 5-HT receptors at a high dose (1.0 mg/kg) [29]. 2-Methyl-5-HT is not a selective 5-HT_3_ receptor agonist but is associated with 5-HT_4_ in pain pathway [30]. However, DOI, a 5-HT_2/1C_, did not affect EAA in this study. These findings suggest that the 5-HT_1A_ and 5-HT_3_ receptors may mediate predominantly EAA elicited at low EAc frequencies. There is evidence that brain serotonergic pathways, involving 5-HT_1_ and 5-HT_3_ receptors, contribute to the antinociceptive effect of EAc that 5-HT_2_ may have a nociceptive function [31]. 

We found that 5,7-DHT (5,7-dihydroxytryptamine) reduced EAA up to 7 days following EAc. It has been reported that 5,7-DHT-induced lesions of the spinal cord serotonergic pathways reduced spinal cord 5-HT concentrations by 70% and notably reduced morphine analgesia as determined by the tail-flick test [32]. The delayed effect of 5,7-DHT on EAA may be related to its neurotoxic effect on serotonergic neuron.

The involvement of mu- and delta-opioid receptors as well as serotonin receptors has been previously described. The present study showed that EAc induced analgesia involvement serotonergic and opioid receptors at the superacute, acute, and subacute stages (1, 3, 7 days) of electroacupuncture-induced analgesia. We also found that responses of different serotonergic and opioid receptor subtypes were associated with electroacupuncture electrical frequencies. Lumbar catheterization of the subarachnoid space in the spine is commonly used in research to study spinal cord functions in rat models which can have a confounding effect on experimental outcomes. Direct lumbar catheterization has several advantages compared with the A-O method, such as decreasing the neurological disturbance and the interference with nociceptive functions of the spinal cord. In the present study, none of the animals died and no detectable signs of neurological impairment were detected after intrathecal catheterization. 

We found that the *μ* opioid receptor participated at three different EAc frequencies, whereas the *δ* receptor was effective at a high EAc frequency (100 Hz). The 5-HT_1A_ and 5-HT_3_ receptors were involved in EAA. 5-HT_1A_ agonist enhanced EAA which was significantly inhibited by 5-HT_1A_ antagonists. We did find that serotonergic and opioid receptors were involved at the superacute, acute, and subacute stage (1, 3, and 7 days) of electroacupuncture analgesia, and those receptors contributed to the antinociceptive effect of EAc. Although the sensitivities of various receptors to the low- and high-frequency EAc are slightly different, the mechanisms of EAA are closely related to the activation of serotonergic and opioid neurons in spinal cord.

## Figures and Tables

**Table 1:** Effects of EAc in different currents on the 1st, 3rd, and 7th days.

	Effect						
Amp	Percentage change of pain threshold						
	Min						
	1st day						
	0 min	15 min	30 min	60 min	90 min	120 min	150 min

Control	−2.34 ± 3.96	−4.91 ± 2.56	−0.89 ± 3.44	−2.32 ± 2.77	−1.62 ± 2.47	−0.41 ± 3.52	2.73 ± 3.50
2 Hz, 1 mA	39.33 ± 5.14	44.60 ± 3.78	51.41 ± 4.49	41.64 ± 4.62	30.12 ± 5.94	30.78 ± 5.21	23.46 ± 3.99
2 mA	25.03 ± 6.88	28.97 ± 7.08	23.65 ± 6.25	25.71 ± 6.56	22.22 ± 3.40	18.55 ± 2.47	20.53 ± 2.67
3 mA	32.76 ± 14.96	9.40 ± 10.64	3.63 ± 11.61	2.57 ± 10.90	4.67 ± 8.96	11.07 ± 8.76	13.23 ± 7.76
10 Hz, 1 mA	30.68 ± 8.00	26.25 ± 7.92	37.47 ± 8.16	26.71 ± 6.39	27.38 ± 6.31	22.34 ± 5.38	20.65 ± 4.03
2 mA	17.30 ± 4.13	25.90 ± 3.78	28.72 ± 9.06	28.51 ± 8.02	26.92 ± 5.16	7.02 ± 3.77	2.37 ± 1.50
3 mA	10.58 ± 17.05	27.77 ± 7.04	13.87 ± 10.54	17.95 ± 9.68	16.40 ± 9.96	16.69 ± 8.80	16.62 ± 7.54
100 Hz, 1 mA	31.34 ± 10.43	22.05 ± 7.83	25.47 ± 8.81	19.96 ± 6.92	19.88 ± 7.69	23.14 ± 11.83	25.30 ± 9.13
2 mA	25.93 ± 8.17	20.27 ± 6.31	21.29 ± 7.47	14.54 ± 5.18	12.20 ± 5.03	2.31 ± 1.88	1.34 ± 3.24
3 mA	10.59 ± 10.34	3.78 ± 15.37	2.43 ± 14.96	−5.91 ± 6.01	−6.49 ± 9.32	−9.36 ± 6.79	−0.25 ± 5.29

	3rd day						
	0 min	15 min	30 min	60 min	90 min	120 min	150 min

Control	1.31 ± 2.74	1.09 ± 3.56	2.27 ± 3.63	0.03 ± 1.76	−1.42 ± 1.86	−0.96 ± 2.02	−0.97 ± 1.51
2 Hz, 1 mA	36.57 ± 5.46	28.70 ± 4.19	29.16 ± 6.49	25.19 ± 7.30	23.48 ± 6.10	19.30 ± 3.56	17.76 ± 4.54
2 mA	16.88 ± 4.01	17.66 ± 5.11	22.42 ± 3.86	11.78 ± 3.80	14.36 ± 3.62	16.53 ± 6.23	11.67 ± 7.02
3 mA	9.47 ± 8.53	4.01 ± 7.86	0.20 ± 9.56	−4.19 ± 6.64	−1.74 ± 5.01	2.98 ± 4.37	3.12 ± 4.07
10 Hz, 1 mA	32.53 ± 12.69	34.92 ± 6.99	61.73 ± 10.60	23.80 ± 5.49	27.27 ± 7.20	10.82 ± 3.75	12.41 ± 4.69
2 mA	22.21 ± 3.65	13.34 ± 5.82	16.51 ± 5.49	9.97 ± 5.26	4.32 ± 2.61	−0.87 ± 2.68	3.30 ± 4.53
3 mA	−5.36 ± 9.16	−3.85 ± 5.27	−5.38 ± 10.82	−9.10 ± 3.63	−6.81 ± 7.40	−2.76 ± 5.30	−2.73 ± 5.44
100 Hz, 1 mA	13.02 ± 9.21	28.95 ± 7.12	25.56 ± 7.96	19.35 ± 5.99	20.21 ± 7.46	16.44 ± 6.57	16.65 ± 6.40
2 mA	10.55 ± 3.38	16.18 ± 5.99	3.64 ± 3.01	5.43 ± 4.38	8.80 ± 3.51	−0.09 ± 3.60	0.69 ± 2.97
3 mA	18.32 ± 5.61	−2.27 ± 7.65	−5.62 ± 5.32	−0.30 ± 4.33	−0.13 ± 3.89	3.04 ± 3.90	3.63 ± 6.23

	7th day						
	0 min	15 min	30 min	60 min	90 min	120 min	150 min

Control	1.28 ± 2.83	1.05 ± 3.74	2.18 ± 3.68	0.05 ± 2.08	−1.38 ± 1.78	−0.94 ± 2.05	−0.94 ± 1.82
2 Hz, 1 mA	35.71 ± 5.33	36.47 ± 3.80	34.76 ± 5.79	35.38 ± 3.75	28.84 ± 3.82	28.08 ± 2.51	26.89 ± 6.12
2 mA	17.47 ± 3.78	12.43 ± 3.22	17.28 ± 3.85	9.98 ± 4.22	8.86 ± 4.91	3.36 ± 2.37	3.08 ± 3.49
3 mA	−1.93 ± 7.94	−4.40 ± 5.65	−2.61 ± 4.41	4.41 ± 6.80	−0.98 ± 3.22	−4.44 ± 4.35	2.06 ± 3.76
10 Hz, 1 mA	36.09 ± 5.90	35.30 ± 4.77	29.37 ± 7.23	29.23 ± 7.24	14.14 ± 3.86	9.52 ± 4.66	10.60 ± 4.23
2 mA	12.49 ± 2.31	8.92 ± 5.82	4.26 ± 5.29	5.07 ± 4.85	2.39 ± 3.71	4.08 ± 5.07	1.20 ± 3.62
3 mA	2.59 ± 1.84	−0.12 ± 5.12	−2.53 ± 3.44	−1.40 ± 3.67	−5.97 ± 4.91	3.89 ± 2.66	7.47 ± 4.41
100 Hz, 1 mA	7.80 ± 3.07	22.35 ± 6.00	29.82 ± 8.99	10.14 ± 3.16	14.32 ± 4.23	9.31 ± 2.89	13.80 ± 3.51
2 mA	8.35 ± 3.43	2.59 ± 1.71	1.72 ± 1.92	5.73 ± 3.48	20.47 ± 3.73	−1.43 ± 2.55	−3.41 ± 2.10
3 mA	25.43 ± 10.43	3.44 ± 5.76	−15.95 ± 4.78	−11.89 ± 4.11	−9.48 ± 2.67	8.59 ± 4.74	6.81 ± 5.90

Data are shown as mean ± S.E.
